# Effects of fertilization gradient on the production performance and nutritional quality of cultivated grasslands in karst areas

**DOI:** 10.3389/fpls.2023.1228621

**Published:** 2023-08-24

**Authors:** Yuefeng Wang, Sihui Tian, Honggang Shuai, Baocheng Jin, Yaoyao Zhang, Junpeng Wei, Zijing Niu, Yifeng Ma, Xuechun Zhao

**Affiliations:** ^1^ College of Animal Science, Guizhou University, Guiyang, China; ^2^ Institute of Botany, Chinese Academy of Sciences, Beijing, China

**Keywords:** organic fertilizer, fertilization gradient, production performance, nutritional quality, cultivated grasslands

## Abstract

Optimal fertilization is an important measure for managing cultivated grasslands, and a necessary means for maintaining the nutrient balance, yield, and quality of grassland ecosystems. This study aimed to explore the effects of organic fertilizers on the production performance and nutritional quality of cultivated grasslands in karst areas. Two types of monocultured cultivated grasslands (i.e., *Medicago sativa* and *Dactylis glomerata*) were employed as the research objects, and a randomized block design was adopted to investigate the effects of five fertilization gradients on the forage height, coverage, yield, and nutritional quality of the cultivated grasslands. According to the results, the plant height, coverage, and yield of *M. sativa* first presented an increasing trend, then decreased with increasing fertilization gradient, with a peak at 20–30 t/hm^2^ fertilization gradient. The height, coverage, and yield of *D. glomerata* increased gradually with increase in fertilization gradient, and peaked at 40 t/hm^2^. Meanwhile, the crude protein (CP) and ether extract (EE) contents of both grassland types displayed first presented an increasing trend, then decreased with increasing fertilization gradient, and peaked at 10–30 t/hm^2^ fertilization gradient. The neutral detergent fiber (NDF) and acidic detergent fiber (ADF) contents of *M. sativa* presented “N-shaped” and “M-shaped” change trends with increasing fertilization gradient, while those of *D. glomerata* showed “V-shaped” and “M-shaped” change trends, reaching minimum values under fertilization gradients of 30 and 20 t/hm^2^, respectively. Year, fertilization, and year × fertilization (Y×F) significantly affected the plant heights, coverages, dry/fresh weight ratios, and yields of *M. sativa* and *D. glomerata*. The contribution of coverage to the subordinate function of *M. sativa* was greatest at a fertilization gradient of 20 t/hm^2^. Meanwhile, the subordinate function values of the height and coverage of *D. glomerata* increased gradually with increasing fertilization gradient, but the difference in the subordinate function value of height was only 0.09%–0.18% under the fertilization gradient of 20–40 t/hm^2^. Evaluation of forage nutrition revealed 10–30 t/hm^2^ and 20–30 t/hm^2^ as the optimal organic fertilizer application rates for *M. sativa* and *D. glomerata*, respectively.

## 1 Introduction

Human demand for livestock products is constantly increasing with the rise of global population, and animal husbandry is facing severe production pressure. High-quality cultivated grasslands form the basis of cattle and sheep breeding and meat production in many regions of the world, playing an important role in global food security and economic benefits (Sanderson et al., 2004). Forage yield and quality are affected by multiple factors, among which fertilization is the most significant (Wang et al., 2010). Reasonable fertilization is an important measure for managing cultivated grasslands, and a necessary means for maintaining the nutrient balance, yield, and quality of grassland ecosystems (Fu et al., 2015). Fertilization can be regulated to maintain the species composition and ensure an ideal yield (Zuzana et al., 2009) and the balance of material input and output in grassland ecosystems, hence achieve sustainable production of the grasslands (Potter et al., 2010; Wang et al., 2013). In China, the insufficient application of organic fertilizers and the unreasonable application of chemical fertilizers are common problems in cultivated grasslands. The application rate of chemical fertilizers is twice the upper limit of safe fertilization, and the input of organic fertilizers only accounts for about 10% of the total nutrients input (Shi et al., 2015). Therefore, fertilization is also key to improving the productivity of cultivated grasslands, besides selecting high-quality and high-yield forage varieties for mixed sowing. Organic fertilizers are rich in organic matter and beneficial microorganisms, and can produce large quantities of organic acids and release the slow-acting nitrogen, phosphorus, and potassium in the soil, which can further effectively alleviate the rapid consumption of soil available nutrients for plant growth, improving soil structure (Ferro et al., 2013), and increasing soil nutrients and moisture conservation capacity. Moreover, they can also enhance the photosynthetic capacity of crops, and further raise crop yield and quality (Beckwith et al., 2002; Marathe et al., 2009; Wang et al., 2012).

Guizhou Province is located in southwestern China, and has a typical karst landform. Rocky desertification in this province is of diverse types and extensively distributed, posing widespread hazards. It is also one of the regions facing the most severe grassland degradation in China (Peng and Wang, 2012; Jiang et al., 2014), seriously restricting the development of grassland animal husbandry. As a major province for animal husbandry in China, Guizhou Province faces the biggest problem of small planting areas for high-yield and high-quality forage, as well as a severe shortage of protein feed and high-quality forage. Thus, establishing high-quality and high-yield cultivated grasslands can effectively mitigate the challenges facing the expansion of animal husbandry in Guizhou Province, maintain the ecological balance of grasslands, and curb further deterioration of fragile grassland ecological environments. *Medicago sativa*, the most important cultivated forage in the world, has a total planting area of 33 million hm² worldwide. Its high yield, good nutritional value and wide adaptability make it known as the “king of forage” (Lamb et al., 2006; Sun et al., 2018). However, *Medicago sativa* is not suitable for cultivation in acid soil in karst region of southwestern China. In order to better develop animal husbandry, the cultivation of *Medicago sativa* will not be delayed. *Dactylis glomerata* was introduced into and cultivated in China in the 1990s, and is currently one of the highest-quality forage varieties suitable for planting throughout the country. It has the advantages of strong tillering ability, large aboveground biomass, abundant leaves, high nutritional value, good palatability, strong adaptability, and stable yield; Both are suitable for the establishment of cultivated grasslands. In this context, the present study employed two types of monocultured cultivated grasslands (i.e., *Medicago sativa* and *Dactylis glomerata*) as the research objects to explore the effects of different fertilization gradients on the production characteristics and nutritional quality of such cultivated grasslands. The research also aimed to identify the optimal fertilization gradient, and thereby provide a theoretical basis for forage production in the cultivated grasslands of karst areas of Guizhou Province.

## 2 Materials and methods

### 2.1 Overview of the study area

The study area is located at Guizhou University in Huaxi District, Guiyang, Guizhou Province (106° 39 ′ E, 26° 27 ′ N), and has an altitude of 1,100 m. It has a subtropical monsoon humid climate, with obvious plateau climate characteristics. The annual average temperature is 15.3°C, the annual rainfall is 1,129.5 mm, the frost-free period is 270 d, while the annual sunshine duration is 1,148.3 h. The soil type is yellow soil. The physical and chemical properties of soil are provided below (**Table S1**). Cultivated grasslands of *Medicago sativa* (cv. 601) and *Dactylis glomerata* (cv. Amba) were established in October 2018. The effects of different fertilization gradients on the production characteristics and nutritional quality were explored from May, 2019 to October, 2022.

### 2.2 Experimental design

*Medicago sativa* (cv. 601) and *Dactylis glomerata* (cv. Amba), provided by Guizhou Zhongzhiheng Ecological Technology Co., Ltd., were used as the experimental materials. Cultivated grasslands of the two varieties were established in October 2018, with a sowing rate of 22.5 kg/hm^2^. The plot size was 2.0 m × 2.0 m, and a drill sowing method was employed. Each plot consisted of six rows, with a row spacing of 30 cm. At the same time, five fertilization gradients (0, 10, 20, 30, and 40 t/hm^2^) were set up in a randomized block design. Organic fertilizer was evenly applied on the soil surface of each plot, and the application was repeated three times. There were 15 plots in each of the cultivated grasslands of *Medicago sativa* and *Dactylis glomerata*. Before sowing, the land was ploughed and prepared, and weeds were removed. After sowing, 2–3 cm of covering soil was provided. Timely field management was ensured throughout the growth period. A sheep manure-fermented organic fertilizer (pH value: 7.4; dry basis nutrient contents: N=2.23%, P_2_O_5 =_ 1.775%, K_2_O=3.91%) was used. Fertilization was done during the early stages of the growing season each year (March 20). Meanwhile, production performance indicators were surveyed during the initial flowering stage of *Medicago sativa* and the heading stage of *Dactylis glomerata* from 2019 to 2022. A 1 m^2^ quadrat was selected in each plot. The survey quadrat was controlled to be 50 cm away from the plot edge to avoid edge effects, and the height (cm), coverage (%), and biomass (g/m^2^) of each species in the quadrat were measured. At maturity, the aboveground parts were cut, brought to the laboratory in bags, dried to a constant weight at 80°C and weighed (g/m^2^). The survey was conducted 5–6 times a year based on growth status. In June, 2019, 2020, 2021 and 2022, the aboveground parts of *Medicago sativa* and *Dactylis glomerata* were harvested, packed in envelopes, brought back to the laboratory, dried at 105°C for 30 min, adjusted to 65°C and dried to a constant weight, and then crushed in a pulverizer and screened with a 40-mesh sieve to determine forage nutrition.

### 2.3 Indicator measurement

Eight main indicators were measured, including plant height, coverage, dry/fresh weight ratio, dry matter yield, crude protein (CP), ether extract (EE), neutral detergent fiber (NDF), and acidic detergent fiber (ADF). The measurement methods were as follows:

Plant height: Randomly select ten plants from each quadrat during sampling, measure the distance from the ground to the highest part of each plant with a tape measure, and calculate the average value as plant height.

Coverage: Record the coverage of forage within each quadrant through visual observation.

Dry/fresh weight ratio and dry matter yield: Immediately weigh fresh forage yield after each cutting, and bring fresh forage to the laboratory in bags. Dry it to a constant weight at 80°C, weigh the dry weight after cooling, and calculate dry/fresh weight ratio.

CP: Kjeldahl nitrogen determination (Jee, 1995).

EE: Soxhlet extraction method (Han et al., 2022), ANKOMAXT15i automatic fat analyzer.

NDF and ADF: measured by the filter bag technology (Mcroberts and Cherney, 2014), using an ANKOM A200i semi-automatic fiber analyzer.

### 2.4 Data processing and statistical analyses

Preliminary data analyses were performed using Office Excel 2019 and one-way ANOVA, while multiple comparisons were conducted using the SPSS 26.0 software. Two-way ANOVA was used to test the significance of the effects of year and fertilization on the production indicators of the two types of cultivated grasslands. Origin 2022 was adopted for drawing. The subordinate function comprehensive evaluation method was employed to comprehensively evaluate the plant heights, coverages, dry/fresh weight ratios, and the grassland yields.

The formula for the subordinate function (Liu et al., 2020) is:


$$U\left(X_{ij}\right)=\frac{X_{ij}-X_{jmin}}{X_{jmax}-X_{jmin}}\left(\text{positive correlation}\right)$$

$$U\left(X_{ij}\right)=1-\frac{X_{ij}-X_{jmin}}{X_{jmax}-X_{jmin}}\left(\text{negative correlation}\right)$$

$$\bar{U}(X_{ij})=\frac{1}{n}\sum_{j=1}^{n}U_{ij}$$

where *U(X_ij_)* denotes the subordinate value of the *j^th^* indicator under the *i^th^* fertilization gradient; *X_ij_* represents the measured value of the *j^th^* indicator under the *i^th^* fertilization gradient; *X_jmax_* and *X_jmin_* denotes the maximum and minimum values of the *j^th^* indicator under all fertilization gradients, respectively;*U(X_ij_)* denotes the average subordinate value of all indicators under the *i^th^* fertilization gradient, that is, the larger the average value, the better the production performance.

## 3 Result analysis

### 3.1 Effects of fertilization on the forage production performance indicators of cultivated grasslands

**Figure 1:**
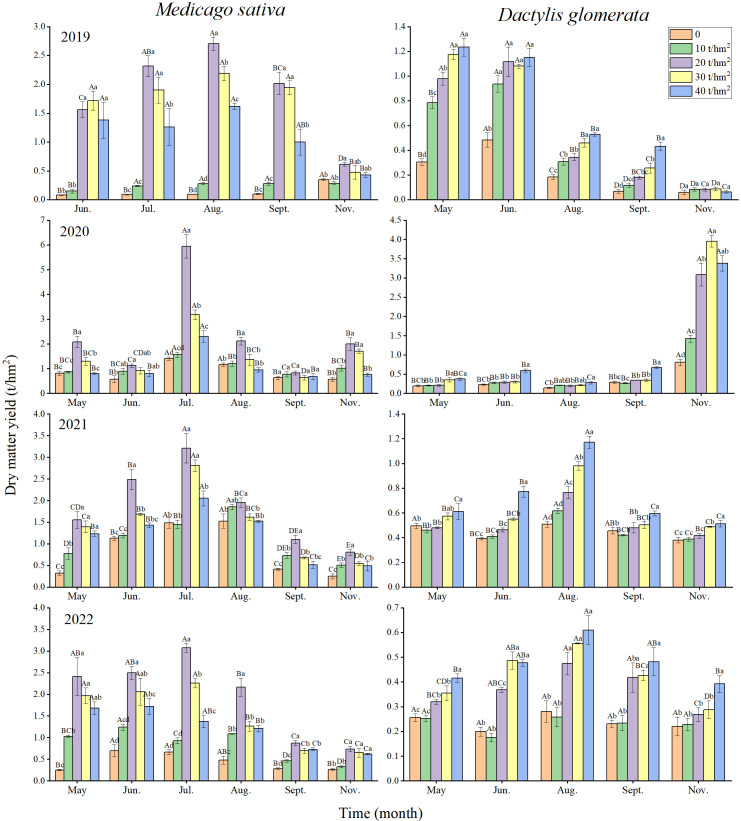
Yield dynamics of *Medicago sativa* and *Dactylis glomerata* in 2019–2022. Different lowercase letters indicate significant differences in different fertilization gradients in the same year, and different uppercase letters indicate significant differences in different years of the same fertilization gradient.

The grasslands of *M. sativa* and *D. glomerata* were surveyed 5–6 times per year from 2019 to 2022. The dry matter yield of *M. sativa* presented a trend of increasing first and decreasing afterward with increasing fertilization gradient, except in November 2019, September 2020, July 2021, and September 2022; and peaked under the fertilization gradient of 20 t/hm^2^, except in June 2019 (**Figure 1**). The seasonal changes in the maximum dry matter yields of *M. sativa* under fertilization also presented a trend of increasing first and decreasing afterward, except in 2020 (**Figure 1**). The maximum values occurred in July or August each year, and were 2.70, 3.21, and 3.08 t/hm^2^, respectively.

The dry matter yield of *D. glomerata* showed a trend of gradual increase with increasing fertilization gradient, except in 2019 and 2020; and peaked under the fertilization gradient of 40 t/hm^2^ (**Figure 1**). In 2019, the maximum dry matter yields of *D. glomerata* under fertilization showed a trend of gradual decrease over time. Conversely, in 2020, the maximum dry matter yields of *D. glomerata* showed a gradual increasing trend. In 2021 and 2022, the maximum dry matter yields of *Dactylis glomerata* presented a trend of increasing first and decreasing afterwards. The maximum values occurred in August, and were 1.17 and 0.61 t/hm^2^, respectively (**Figure 1**).The annual dry matter yield of *M. sativa* presented a trend of increasing first and decreasing afterward with increasing fertilization gradient in 2019–2022, and peaked under the fertilization gradient of 20 t/hm^2^ (**Table 1**). The maximum values were 8.14, 13.87, 11.85, and 13.08 t/hm^2^, respectively, and the maximum values were 2.17–12.72 times the minimum values (under the fertilization gradient of 0 t/hm^2^). From 2019 to 2022, the annual dry matter yield of *M. sativa* presented a trend of increasing first and decreasing afterward under the same fertilization gradient. Under the fertilization gradients of 0 and 10 t/hm^2^, the maximum annual dry matter yields of *Medicago sativa* all occurred in 2021, and were 5.47 and 6.94 t/hm^2^, respectively. Under the fertilization gradients of 20, 30, and 40 t/hm^2^, the annual dry matter yield of *Medicago sativa* showed a trend of gradual increase under the same fertilization gradient, and the maximum values all occurred in 2022 and were 13.08, 9.90, and 8.16 t/hm^2^, respectively (**Table 1**).

**Table 1:** Annual dry matter yield (t/hm^2^) of forage under different fertilization gradients.

Species	Year	Fertilization gradients(t/hm^2^)					
		0	10	20	30	40	AVE
*Medicago sativa*	2019	0.64 ± 0.02^Dc^	1.09 ± 0.002^Dc^	8.14 ± 0.36^Ba^	7.26 ± 0.59^Ba^	5.03 ± 0.73^Bb^	4.43 ± 0.18
	2020	5.06 ± 0.04^Bc^	6.20 ± 0.22^Bc^	13.87 ± 0.94^Aa^	8.95 ± 0.63^Ab^	6.19 ± 0.29^Bc^	8.05 ± 0.34
	2021	5.47 ± 0.05^Ad^	6.94 ± 0.16^Acd^	11.85 ± 0.98^Aa^	9.30 ± 0.34^Ab^	7.72 ± 0.32^Ac^	8.26 ± 0.45
	2022	2.94 ± 0.23^Ce^	5.64 ± 0.09^Cd^	13.08 ± 0.69^Aa^	9.90 ± 0.37^Ab^	8.16 ± 0.23^Ac^	7.94 ± 0.35
	AVE	3.53 ± 0.08	4.97 ± 0.11	11.73 ± 0.68	8.85 ± 0.22	6.77 ± 0.12	7.17
*Dactylis glomerata*	2019	1.06 ± 0.10^Ce^	2.15 ± 0.10^Ad^	2.60 ± 0.13^Bc^	2.95 ± 0.03^Bb^	3.29 ± 0.11^Ba^	2.14 ± 0.09
	2020	1.60 ± 0.14^Bc^	2.28 ± 0.06^Ac^	3.97 ± 0.31^Ab^	4.97 ± 0.22^Aa^	5.10 ± 0.29^Aa^	3.58 ± 0.07
	2021	2.15 ± 0.04^Ad^	2.21 ± 0.04^Ad^	2.51 ± 0.08^Bc^	2.98 ± 0.07^Bb^	3.53 ± 0.08^Ba^	2.67 ± 0.23
	2022	1.14 ± 0.08^Cd^	1.10 ± 0.04^Bd^	1.78 ± 0.12^Cc^	2.03 ± 0.05^Cb^	2.29 ± 0.06^Ca^	1.67 ± 0.05
	AVE	1.49 ± 0.06	1.94 ± 0.04	2.72 ± 0.08	3.23 ± 0.06	3.55 ± 0.11	2.58

Different lowercase letters indicate significant differences in different fertilization gradients in the same year, and different uppercase letters indicate significant differences in different years of the same fertilization gradient.

The annual dry matter yield of *D. glomerata* showed a trend of gradual increase with increasing fertilization gradient in 2019–2022, and peaked under the fertilization gradient of 40 t/hm^2^. The maximum values were 3.29, 5.10, 3.53, and 2.29 t/hm^2^, respectively, and the maximum values were 1.64–3.19 times of the minimum values (under the fertilization gradients of 0 and 10 t/hm^2^) (**Table 1**).

**Figure 2:**
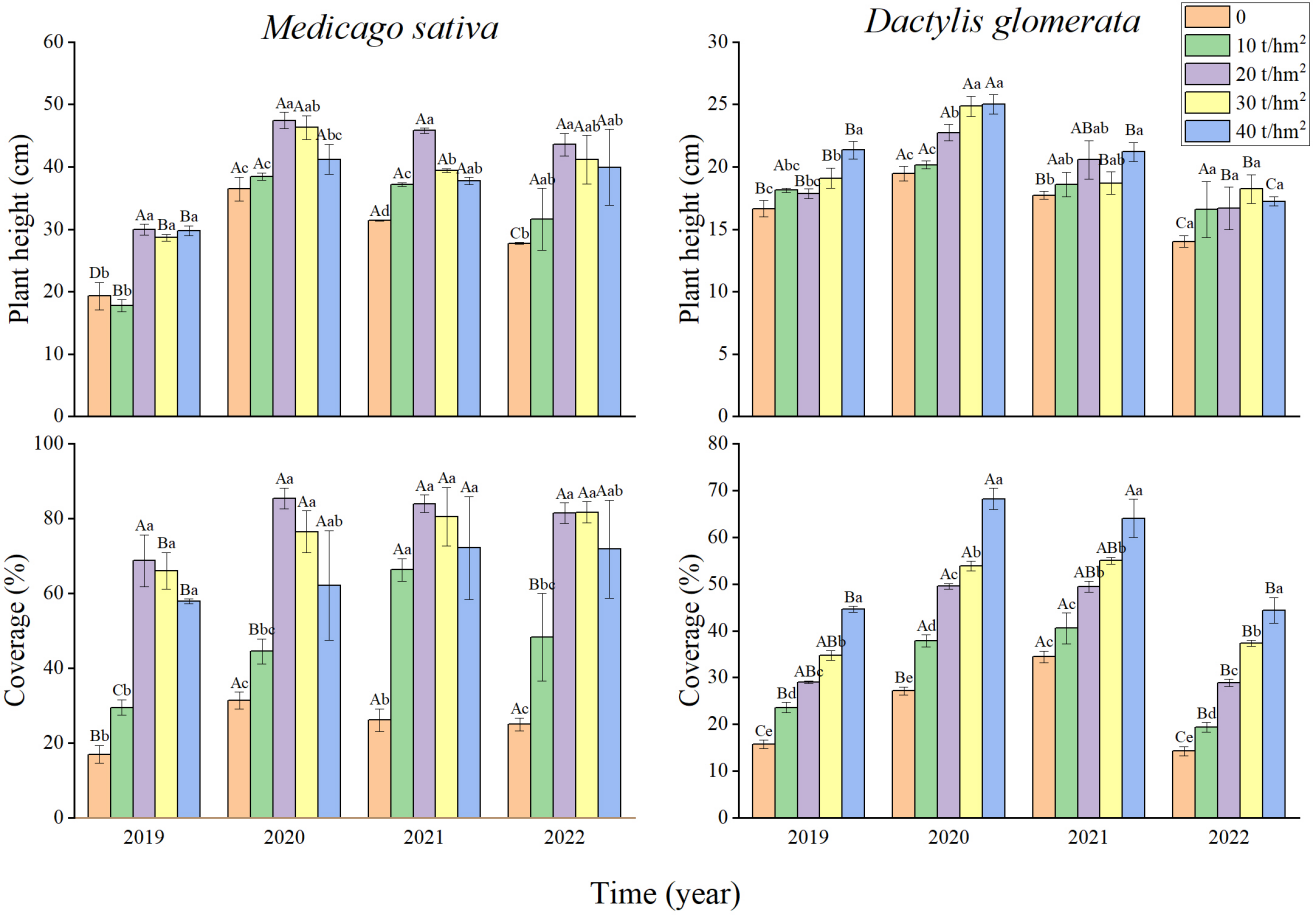
Dynamics of plant height, coverage and dry/fresh ratio of *Medicago sativa* and *Dactylis glomerata* under different fertilization gradients. Different lowercase letters indicate significant differences in different fertilization gradients in the same year, and different uppercase letters indicate significant differences in different years of the same fertilization gradient.

**Figure 2** shows that the annual average plant height of *M. sativa* showed a trend of gradual increase, except in 2019. Annual average plant height and coverage both presented a trend of increasing first and decreasing afterwards with increasing fertilization gradient within the four years, and both peaked under the fertilization gradient of 20 t/hm^2^. The maximum annual plant heights were 1.38–2.16 times the minimum annual plant heights. The maximum annual coverages were 1.22–2.24 times the minimum annual coverages.

From 2019 to 2022, the annual average plant height of *D. glomerata* showed a gradual increase. The maximum annual plant heights (19.46, 20.16, 22.75, 24.85, and 25.01 cm) were 1.22–1.45 times the minimum annual plant heights. The annual average coverage of *D. glomerata* showed a trend of gradual increase with increasing fertilization gradient. The maximum annual coverages (34.47%, 40.53%, 49.4%, 55.00%, and 64.00%, respectively) of *D. glomerata* were 1.44–2.18 times the minimum annual coverages (**Figure 2**).

### 3.2 Effects of fertilization on the forage nutritional quality of cultivated grasslands

**Table 2** shows that the CP and EE contents of *M. sativa* both presented a trend of increasing first and decreasing afterward with increasing fertilization gradient, and peaked under the fertilization gradients of 30 and 10 t/hm^2^, respectively. The maximum values (21.33% and 11.34%) were 1.42 and 1.43 times of the minimum values. The NDF and ADF contents of *M. sativa* presented “N-shaped” and “M-shaped” change trends with increasing fertilization gradient, and reached their minimum values under the fertilization gradients of 30 and 20 t/hm^2^, respectively. Their minimum values (22.62% and 30.95%) were 72.80% and 94.13% of their maximum values, respectively.

**Table 2:** Changes in the forage nutritional quality of the two types of cultivated grasslands under different fertilization gradients.

Species	Index	Fertilization gradients(t/hm^2^)				
		0	10	20	30	40
*Medicago sativa*	CP/%	15.02 ± 1.21^d^	17.96 ± 1.27^c^	21.23 ± 1.31a	21.33 ± 1.26^a^	20.97 ± 1.22^b^
	EE/%	8.30 ± 1.39^ab^	11.34 ± 0.44^a^	11.03 ± 0.76a	9.65 ± 1.20^ab^	7.93 ± 0.66^b^
	NDF/%	24.73 ± 2.00^bc^	31.07 ± 2.38^a^	26.30 ± 2.68b	22.62 ± 3.36^c^	23.23 ± 3.11^c^
	ADF/%	32.76 ± 3.91^a^	32.88 ± 3.47^a^	30.95 ± 3.41b	32.01 ± 4.06^ab^	31.52 ± 3.21^ab^
*Dactylis glomerata*	CP/%	15.88 ± 1.29^c^	16.96 ± 1.23^b^	18.04 ± 1.22^a^	17.99 ± 1.21^a^	17.95 ± 1.32^a^
	EE/%	2.23 ± 0.10^b^	2.56 ± 0.43^b^	3.63 ± 0.14^a^	3.76 ± 0.17^a^	3.62 ± 0.28^a^
	NDF/%	36.14 ± 1.27^a^	35.95 ± 2.60^a^	35.47 ± 2.29^a^	33.37 ± 1.90^a^	34.20 ± 1.56^a^
	ADF/%	30.62 ± 2.48^a^	31.79 ± 2.03^a^	30.46 ± 2.41^a^	31.88 ± 2.33^a^	31.52 ± 2.59^a^

Different letters indicate significant differences. CP, crude protein; EE, ether extract; NDF, neutral detergent fiber; ADF, acidic detergent fiber.

Similar to *M. sativa*, the CP and EE contents of *D. glomerata* also presented a trend of increasing first and decreasing afterward with increasing fertilization gradient, and peaked under the fertilization gradients of 20 and 30 t/hm^2^, respectively (**Table 2**). The maximum values (18.04% and 3.76%) were 1.14 and 1.67 times of the minimum values. The NDF and ADF contents of *D. glomerata* presented “V-shaped” and “M-shaped” change trends with increasing fertilization gradient, and peaked under the fertilization gradients of 30 and 20 t/hm^2^, respectively. Their minimum values (33.37% and 30.46%) were 92.34% and 95.55% of their maximum values, respectively.

### 3.3 Relationships between fertilization and the forage production performance of cultivated grasslands

According to the two-way ANOVA (**Table 3**) of forage production performance indicators using fertilization and year, year, fertilization, and Y × F all significantly affected the plant height, coverage, dry/fresh ratio, and yield of *M. sativa* (*P*<0.05), except that Y × F had a non-significant effect on coverage. Year, fertilization, and year × fertilization (Y×F) all significantly affected the plant height, coverage, dry/fresh ratio, and yield of *D. glomerata* (*P*<0.05), except that Y × F had a non-significant effect on plant height (*P*>0.05).

**Table 3:** Results (F-values) of an ANOVA of fertilization and year and their interactions on the plant heights, coverages, dry/fresh ratios, and yields (DMY) of *Medicago sativa* and *Dactylis glomerata*.

index	*Medicago sativa*			*Dactylis glomerata*		
	Year(Y)	Fertilization(F)	Y×F	Year(Y)	Fertilization(F)	Y×F
Plant height	51.54**	22.57**	1.00**	32.59**	11.77**	1.28
Coverage	6.26**	43.71**	0.66	218.89**	236.05**	2.10*
Dry/Fresh	120.86**	10.70**	8.50**	97.93**	25.53**	13.65**
DMY	44.67**	189.39**	6.31**	175.84**	168.48**	16.28**

**Figure 3:**
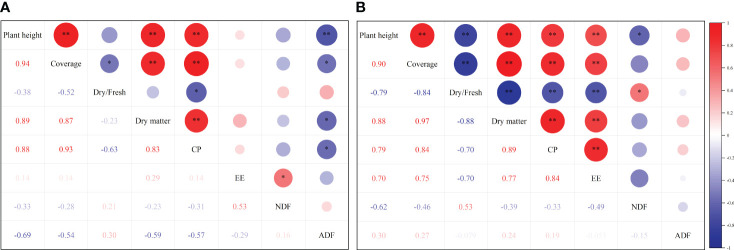
Correlation analysis of production indicators under different fertilization gradients. **(A)** *Medicago sativa*; **(B)** *Dactylis glomerata*. ANOVA, analysis of variance. * *P*<0.05.** *P*<0.01.

According to the correlation analysis between the forage indicators of the cultivated grasslands of *M. sativa* and *D. glomerata* (**Figure 3**), the plant height of *M. sativa* was significantly positively correlated with coverage, yield, and CP content and significantly negatively correlated with ADF content (*P*<0.05), but had no significant correlation with dry/fresh ratio, EE content, or NDF content (*P*>0.05). The coverage of *M. sativa* was significantly negatively correlated with dry/fresh ratio and ADF content and significantly positively correlated with yield and CP content (*P*<0.05). The EE content of *M. sativa* was significantly positively correlated with NDF content (*P*<0.05), but had no significant correlation with any other indicator (*P*>0.05) (**Figure 3A**).

The coverage, dry matter yield, CP content, and EE content of *D. glomerata* were significantly positively correlated (*P*<0.05). The plant height of *D. glomerata* was significantly negatively correlated with dry/fresh ratio and NDF content (*P*<0.05). The dry/fresh ratio of *D. glomerata* was significantly positively correlated with NDF content, and significantly negatively correlated with plant height, dry matter yield, CP, and EE (*P*<0.05). The ADF content of *D. glomerata* had no significant correlation with any other indicator (*P*>0.05) (**Figure 3B**).

Comprehensive evaluation of the subordinate function based on height, coverage, dry/fresh ratio, and yield (**Table 4**) indicated that the average subordinate function values of *M. sativa* under the fertilization gradient of 20 t/hm^2^ were all greater than those under other fertilization gradients. In 2019–2022, under the fertilization gradients of 0, 10, and 40 t/hm^2^, the average subordinate function values of *M. sativa* all presented a trend of increasing first and decreasing afterward over time, and peaked in 2020, 2021, and 2021, with maximum values of 0.41, 0.55, and 0.70, respectively. Under the fertilization gradient of 20 t/hm^2^, the average subordinate function values of *M. sativa* presented a trend of increasing first, decreasing afterward, and then increasing again over time, and peaked in 2022, with a maximum value of 0.83. Under the fertilization gradient of 30 t/hm^2^, the average subordinate function values of *M. sativa* kept increasing over time, and peaked in 2022, with a maximum value of 0.76.

**Table 4:** Comprehensive evaluation of subordinate function based on forage production performance of the two types of cultivated grasslands under different fertilization gradients.

Fertilization (t/hm^2^)	Year	*Medicago sativa*					*Dactylis glomerata*								
		Plant height	Coverage	Dry/Fresh	Dry matter	Average	Rank		Plant height	Coverage	Dry/Fresh	Dry matter	Average	Rank	
0	2019	0.12	0.03	0.06	0.00	0.05	20		0.27	0.05	0.28	0.03	0.16	19	
	2020	0.60	0.22	0.50	0.31	0.41	15		0.48	0.24	0.46	0.15	0.33	13	
	2021	0.46	0.15	0.46	0.33	0.35	17		0.35	0.36	0.20	0.27	0.29	16	
	2022	0.36	0.14	0.66	0.16	0.33	18		0.07	0.02	0.18	0.05	0.08	20	
10	2019	0.08	0.19	0.36	0.03	0.17	19		0.38	0.18	0.38	0.27	0.30	14	
	2020	0.66	0.38	0.44	0.38	0.47	13		0.53	0.42	0.35	0.29	0.40	9	
	2021	0.62	0.66	0.47	0.44	0.55	9		0.41	0.46	0.01	0.28	0.29	17	
	2022	0.47	0.43	0.74	0.35	0.49	11		0.26	0.11	0.28	0.04	0.17	18	
20	2019	0.42	0.69	0.27	0.52	0.48	12		0.42	0.27	0.51	0.36	0.39	10	
	2020	0.95	0.90	0.47	0.91	0.81	2		0.83	0.61	0.33	0.65	0.61	4	
	2021	0.80	0.88	0.38	0.77	0.71	5		0.54	0.61	0.21	0.34	0.43	8	
	2022	0.87	0.85	0.74	0.86	0.83	1		0.40	0.27	0.33	0.19	0.30	15	
30	2019	0.38	0.66	0.13	0.46	0.41	14		0.52	0.37	0.77	0.44	0.52	6	
	2020	0.84	0.79	0.45	0.57	0.66	7		0.89	0.69	0.45	0.87	0.72	2	
	2021	0.68	0.84	0.83	0.60	0.74	4		0.48	0.71	0.27	0.44	0.47	7	
	2022	0.76	0.85	0.79	0.64	0.76	3		0.34	0.41	0.39	0.24	0.35	12	
40	2019	0.44	0.55	0.19	0.30	0.37	16		0.57	0.53	0.94	0.51	0.64	3	
	2020	0.68	0.61	0.51	0.38	0.54	10		0.84	0.93	0.32	0.89	0.74	1	
	2021	0.64	0.73	0.93	0.49	0.70	6		0.55	0.86	0.17	0.56	0.53	5	
	2022	0.55	0.73	0.81	0.52	0.65	8		0.29	0.53	0.34	0.30	0.36	11	

The average subordinate function values of *D. glomerata* under the 40 t/hm^2^ fertilization gradient were all greater than those under other fertilization gradients. In 2019–2022, the average subordinate function values of *D. glomerata* all presented a trend of increasing first and decreasing afterward over time. The average subordinate function values under the five fertilization gradients all peaked in 2020, with maximum values of 0.33, 0.40, 0.61, 0.72, and 0.74, respectively.

## 4 Discussion

### 4.1 Effects of fertilization on forage height and coverage

The establishment and management modes of cultivated grasslands have greatly changed the biological and abiotic factors of grassland ecosystems, and modified their energy and material circulation patterns. Fertilization is one of the most direct and effective management modes of cultivated grasslands. For cultivated grasslands, forage height and coverage significantly correlate with forage yield and species. In this study, the height and yield of the grassland of *M. sativa* were both significantly higher than those of the grassland of *D. glomerata*. The main reason is that *M. sativa* is an erect-type top-grass, while *D. glomerata* is a rhizomatous top-grass, leading to significant differences in forage height and yield between different types of cultivated grasslands. Reasonable fertilization can increase the forage height and coverage of grasslands. In this study, the plant height and coverage of *M. sativa* both peaked under the fertilization gradient of 20 t/hm^2^, and were 30.14%–57.16% and 172.04%–304.41% higher than those of untreated *M. sativa*, respectively. The plant height and coverage of *D. glomerata* gradually increased with increasing fertilization gradient, both peaked under the fertilization gradient of 40 t/hm^2^, and were 19.64%–28.55% and 85.69%–211.21% higher than those of untreated *D. glomerata*, respectively. These findings are consistent with a previous study (Zhou et al., 2005), which also found that the forage height and coverage of cultivated grasslands increased by 15.40%–83.10% and 10.60%–174.0% after fertilization, respectively. Based on the results from comprehensive evaluation of subordinate function (**Table 4**), the contribution of coverage to the subordinate function of *M. sativa* was the greatest under the fertilization gradient of 20 t/hm^2^, suggesting that 20 t/hm^2^ is the optimal organic fertilizer application rate for *M. sativa*. The subordinate function values of the height and coverage of *D. glomerata* showed a trend of gradual increase with increasing fertilization gradient. However, under the fertilization gradient of 20–40 t/hm^2^, the difference in the subordinate function value of height was only 0.09%–0.18%, and that in the subordinate function value of coverage was only 19.27%–23.51%. In terms of fertilization costs, 20 t/hm^2^ is likely to be the optimal organic fertilizer application rate for *D. glomerata* as well.

### 4.2 Effects of fertilization on forage yield and quality

Fertilization has the most significant effect on promoting the growth and development, yield increase, and income increase of forage. It boosts the tillering of *D. glomerata* and *M. sativa*, and increases the dry matter weights of aboveground and underground parts. Fertilizer application rate is positively correlated with forage yield and quality within a certain range of fertilization, but yield begins to decrease beyond the critical application rate of the organic fertilizer. In this study, the critical application rate of the organic fertilizer for *M. sativa* was 20 t/hm^2^. The critical application rate for *D. glomerata* was greater than 40 t/hm^2^, under which the yield-increasing effect on *D. glomerata* was already weaker than that under a low fertilization gradient. Thus, the critical application rate for *D. glomerata* should be around 40 t/hm^2^. In this study, the critical application rate of the organic fertilizer for *M. sativa* was lower than that for *D. glomerata*, mainly because legume plants can coexist with rhizobia and provide a large amount of nitrogen, which reduces the demand of *M. sativa* for the organic fertilizer. Bai and Qiu et al. identified the nitrogen application rate of 105–300 kg/hm^2^ per year as the threshold of mature or degraded grasslands, and that, beyond this application rate, changes in grassland biomass, species diversity, and functional group structure would no longer be obvious (Qiu and Luo, 2004; Bai et al., 2010). In this study, the critical application rates of the organic fertilizer for *M. sativa* and *D. glomerata* could be converted to nitrogen at 446 and 892 kg/hm^2^, which were much higher than the threshold of nitrogen addition. This can be explained from two aspects: First, the cultivated grasslands in this study were newly cultivated land with low soil nutrients (**Table S1**). Second, cultivated grasslands consume a lot of soil nutrients due to continuous cutting and output, thus only a high application rate could offset the absorption of soil nutrients by forage. The yields of *M. sativa* and *D. glomerata* fluctuated significantly between years. The yield of *M. sativa* was low only in 2019, but remained at a high level over the next three years. In 2019, the first year after the establishment of the grassland of *M. sativa*, fertilization preferentially promoted root growth, and most nutrients were allocated to roots, thereby accelerating the establishment of fine root meristems and roots (Beck, 1996; Takei et al., 2001). As a result, the growth of aboveground parts lagged behind that of roots. Meanwhile, the yield of *D. glomerata* was low in 2019 and lowest in 2022, which might be related to the climate anomaly of that year. That is, the summer of 2022 had a higher temperature and less rainfall relative to previous years, and, when the temperature was above 28°C, the growth and tillering of *D. glomerata* slowed down (Zhang et al., 2019). Moreover, *D. glomerata* is a shallow-rooted forage, the yield of which responds significantly to temperature rise and drought (Thivierge et al., 2016).

Nutrition quality is the basis for evaluating the quality of forage, and nutrient content is one of the most important indicators to consider while measuring the feeding value of forage. CP and EE contents largely determine the nutritional quality of forage (Hu et al., 2014). In this study, the CP and EE contents of *M. sativa* and *D. glomerata* presented a trend of increasing first and decreasing afterward with increasing fertilization gradient. The CP and EE contents after fertilization were significantly higher than those without fertilization. Under the fertilization gradient of 10–30 t/hm^2^, the CP and EE contents of *M. sativa* peaked, while the NDF and ADF contents reached their minimum values. In terms of forage nutrition, the optimal organic fertilizer application rate for *M. sativa* should be 10–30 t/hm^2^. *D. glomerata* had the highest CP and EE contents and the lowest NDF and ADF contents under the fertilization gradient of 20–30 t/hm^2^. In terms of forage nutrition, the optimal organic fertilizer application rate for *D. glomerata* should be 20–30 t/hm^2^. In practical production, fertilizer application rate depends on the goal pursued. For example, a moderate application rate can be considered if economic benefits are taken as the goal, whereas a high application rate can be implemented if yield is the goal.

## 5 Conclusions

This study investigated the responses of the monocultured cultivated grasslands of *M. sativa* and *D. glomerata* to fertilization gradients, to determine their optimal application rates. To sum up, the dry matter yield of *M. sativa* presented the same trend (i.e., increasing first and decreasing afterward) with increasing fertilization gradient across different years from 2019 to 2022, and peaked under the fertilization gradient of 20 t/hm^2^. The dry matter yield of *D. glomerata* increased with increasing fertilization gradient, and peaked under the fertilization gradient of 40 t/hm^2^. The yield-increasing effect was weakened under the fertilization gradient of 30 t/hm^2^, suggesting that there is a threshold of nitrogen addition for the yields of the monocultured cultivated grasslands of *M. sativa* and *D. glomerata*. For *M. sativa* and *D. glomerata*, dry matter yield, plant height, and coverage had highly significant positive correlations with each other, and highly significant negative correlations with dry/fresh ratio. NDF and ADF contents had weak correlations with other indicators. Comprehensive evaluation of subordinate function indicated that the evaluation effect of *M. sativa* was the best under the fertilization gradient of 20 t/hm^2^, and that the evaluation effect of *D. glomerata* was the best under the fertilization gradient of 20 t/hm^2^. Therefore, reasonable organic fertilizer application should avoid fertilizer wastage and maximize the economic benefits. This study set the optimal fertilizer application rate at 20 t/hm^2^ for the monocultured artistic grasslands of *M. sativa*, and at 40 t/hm^2^ for the monocultured artistic grasslands of *D. glomerata*.

## Data availability statement

The raw data supporting the conclusions of this article will be made available by the authors, without undue reservation.

## Author contributions

Conceptualization, YW, XZ and BJ; methodology, YW, XZ and ST; software, YW, XZ and ST; validation, YW, YZ, XZ, and HS; investigation, YW, XZ, JW, ZN, and YM; writing—original draft preparation, YW, BJ and XZ. All authors have read and agreed to the published version of the manuscript.
