# Supplementary material for: Effects of fertilization gradient on the production performance and nutritional quality of cultivated grasslands in karst areas
Source: Front Plant Sci. 2023 Aug 24;14:1228621. doi: 10.3389/fpls.2023.1228621 (PMC10484102; doi:10.3389/fpls.2023.1228621)
Supplement: Supplementary file 1 [file Table_1.docx]

**Table S1.** Physical and chemical properties of soil in the experimental site

| Study area | pH | Alkali-hydrolyzable nitrogen, mg/kg | Total nitrogen, mg/kg | Total potassium, g/kg | Total phosphorus, g/kg | Rapidly available potassium, mg/kg | Organic carbon, g/kg | Available phosphorus, mg/kg | Available manganese, mg/kg | Available iron, mg/kg | Available zinc, mg/kg |
| --- | --- | --- | --- | --- | --- | --- | --- | --- | --- | --- | --- |
| Experimental site | 5.84 | 33.32 | 655 | 26.13 | 0.76 | 293.33 | 5.68 | 4.74 | 9.68 | 34.77 | 1.34 |
